# Spontaneous Brain Activity in the Default Mode Network Is Sensitive to Different Resting-State Conditions with Limited Cognitive Load

**DOI:** 10.1371/journal.pone.0005743

**Published:** 2009-05-29

**Authors:** Chaogan Yan, Dongqiang Liu, Yong He, Qihong Zou, Chaozhe Zhu, Xinian Zuo, Xiangyu Long, Yufeng Zang

**Affiliations:** 1 State Key Laboratory of Cognitive Neuroscience and Learning, Beijing Normal University, Beijing, China; 2 Institute for Pediatric Neuroscience, New York University Child Study Center, New York, New York, United States of America; University of Granada, Spain

## Abstract

**Background:**

Recent functional MRI (fMRI) studies have demonstrated that there is an intrinsically organized default mode network (DMN) in the resting brain, primarily made up of the posterior cingulate cortex (PCC) and the medial prefrontal cortex (MPFC). Several previous studies have found that the DMN is minimally disturbed during different resting-state conditions with limited cognitive demand. However, this conclusion was drawn from the visual inspection of the functional connectivity patterns within the DMN and no statistical comparison was performed.

**Methodology/Principal Findings:**

Four resting-state fMRI sessions were acquired: 1) eyes-closed (EC) (used to generate the DMN mask); 2) EC; 3) eyes-open with no fixation (EO); and 4) eyes-open with a fixation (EO-F). The 2–4 sessions were counterbalanced across participants (n = 20, 10 males). We examined the statistical differences in both functional connectivity and regional amplitude of low frequency fluctuation (ALFF) within the DMN among the 2–4 resting-state conditions (i.e., EC, EO, and EO-F). Although the connectivity patterns of the DMN were visually similar across these three different conditions, we observed significantly higher functional connectivity and ALFF in both the EO and the EO-F conditions as compared to the EC condition. In addition, the first and second resting EC conditions showed significant differences within the DMN, suggesting an order effect on the DMN activity.

**Conclusions/Significance:**

Our findings of the higher DMN connectivity and regional spontaneous activities in the resting state with the eyes open suggest that the participants might have more non-specific or non-goal-directed visual information gathering and evaluation, and mind wandering or daydreaming during the resting state with the eyes open as compared to that with the eyes closed, thus providing insights into the understanding of unconstrained mental activity within the DMN. Our results also suggest that it should be cautious when choosing the type of a resting condition and designating the order of the resting condition in multiple scanning sessions in experimental design.

## Introduction

Functional neuroimaging studies have consistently demonstrated that some brain regions show task-independent deactivation across a wide range of cognitive tasks compared with the baseline states (e.g., resting-state) [1]–[3]. These brain regions include the posterior cingulate cortex (PCC), precuneus (PCu), medial prefrontal cortex (MPFC), ventral anterior cingulate cortex (vACC), and lateral parietal cortex. They are considered to reflect a default mode of brain function [4] and to constitute a default-mode network (DMN) [5]. Recent research has suggested that the DMN might be associated with the collection and evaluation of information [6], the self-referential mental activity [7], the extraction of episodic memory [8], emotion and anxiety [9], [10], and mind wandering or daydreaming [11].

The DMN has been observed by many resting-state functional magnetic resonance imaging (fMRI) studies [5], [12]–[17] since Biswal and colleagues [18] reported that the low frequency (0.01–0.08 Hz) fluctuations (LFFs) of the resting-state fMRI signal were of physiological importance. The LFFs of resting-state fMRI signal were suggested to reflect spontaneous neuronal activity [19], [20]. And The LFFs within the DMN are highly synchronized among regions (i.e., functional connectivity) during several different resting conditions, such as the awake resting state [5], [14], [15], light sleep in humans [21], and even during an anaesthetized state in monkeys [22]. Several fMRI studies demonstrated that these functional connectivity patterns were minimally disturbed across different resting-state conditions with limited cognitive demand. For example, Greicius and colleagues [5] observed that the functional connectivity patterns of the DMN in the eyes-closed resting state were virtually identical to that in a passive visual processing task. Fox and colleagues [14] found that the functional connectivity patterns of the DMN were strikingly similar across three different resting states (visual fixation, eyes-closed, and eyes-open). Fransson [15] found that the functional connectivity patterns of the DMN were very similar during the eyes-closed and eyes-open states. This finding suggested that self-reflective thoughts, monitoring of the environment, and emotional states could be at work in the two conditions. By visual inspection of functional connectivity patterns of the DMN, all these studies suggested that the connectivity patterns of the DMN were quite similar across different resting states. However, until now, there is no study quantitatively examined the differences in functional connectivity of the DMN among different resting conditions. Such a study would add further understanding on unconstrained mental activity within the DMN.

While functional connectivity analysis measures the signal synchrony of LFF activity among different brain areas, it does not provide information of regional spontaneous activity. The regional activities during rest can be examined by several metrics, such as the root mean square [18], [23], the power spectrum [24], [25], the amplitude of low frequency fluctuation (ALFF) [26], [27], the low-frequency spectral amplitude [28], and the resting state physiological fluctuation amplitude [29]. It would also be vital to explore whether there are significant differences in the regional spontaneous activities in the DMN among the different resting-state conditions.

In considering the clinical studies examining DMN of cognitive disorders, different resting-state conditions have been used [30]–[37]. For example, in attention-deficit/hyperactivity disorder (ADHD) studies, some used the eyes-open condition [35], [36], while others used the eyes-closed condition [37]. If there are significant differences in the spontaneous activity of the DMN across different resting conditions, it should be cautious when choosing the type of a resting condition and comparing the results from different studies in which different resting-state conditions were used.

To address these issues, we examined the statistical differences in both the functional connectivity and the regional ALFF within the DMN across three different conditions: eyes-closed (EC), eyes-open with no fixation (EO), and eyes-open with a fixation (EO-F).

## Materials and Methods

### Participants and Experiments

Twenty healthy right-handed college students (10 females, 10 males, 21.0±1.82 years, ranging from 18 to 24) participated in this study. They had no history of neurological and psychiatric disorders and never participated in any MRI experiment before. Written informed consent was obtained from each participant, and this study was approved by the Institutional Review Board of Beijing Normal University Imaging Center for Brain Research. There were totally six scanning sessions for each participant. Briefly, the participants first underwent an EC resting-state scan session. This session was used to create the midline regions of interest (ROIs) in the DMN (See *Generation of Midline ROIs* below). Then, the following four sessions, three for resting-state and one for task state, were acquired and counterbalanced across the participants: 1) EC, 2) EO, 3) EO-F, and 4) a fast event-related visual response task. Finally, there was a block-designed task session. Each of the six sessions lasted for eight minutes. During the three resting-state sessions, the participants were instructed to keep as motionless as possible and not to think systematically. During the EO-F session, the participants were instructed to fixate on the black crosshair in the center of a white screen. Immediately after each scanning session, the experiment operator had a short communication with the participants. All participants reported that they had not fallen asleep during the scan. The two task-related sessions (event-related and block-designed) were not used in the current study.

### Image Acquisition

MRI data were acquired using a SIEMENS TRIO 3-Tesla scanner in the Beijing Normal University Imaging Center for Brain Research. The participants lay supine with the head snugly fixed by straps and foam pads to minimize head movement. The functional images were obtained using an echo-planar imaging sequence with the following parameters: 33 axial slices, thickness/gap = 3/0.6 mm, in-plane resolution = 64×64, TR = 2000 ms, TE = 30 ms, flip angle = 90°, FOV = 200×200 mm. In addition, a T1-weighted sagittal three-dimensional magnetization-prepared rapid gradient echo (MPRAGE) sequence was acquired, covering the entire brain: 128 slices, TR = 2530 ms, TE = 3.39 ms, slice thickness = 1.33 mm, flip angle = 7°, inversion time = 1100 ms, FOV = 256×256 mm, and in-plane resolution = 256×192.

### Preprocessing of Functional Data

Four resting-state sessions were analyzed in the current study: the first eyes-closed session was used to create the midline ROIs in the DMN, and the three other sessions, EC, EO, and EO-F were used to compare between-condition activities within the midline ROIs. The first 10 volumes of the functional images were discarded for the signal equilibrium and participants' adaptation to the scanning noise. The slice timing, head motion correction, and spatial normalization with re-sampling to 3×3×3 mm were conducted by using *Statistical Parametric Mapping* (SPM2, http://www.fil.ion.ucl.ac.uk/spm). No participant had head motion of more than 2.0 mm maximum displacement in any of the x, y, or z directions nor 2.5° of any angular motion throughout the course of scan. An in-house software, *Resting-State fMRI Data Analysis Toolkit* (REST, by SONG Xiaowei, YAN Chaogan et al., http://resting-fmri.sourceforge.net), was then used for removing the linear trend of time courses and for temporally band-pass filtering (0.01–0.08 Hz) [18], [38]. The resulting data was transformed to the Talairach atlas [39] and spatially smoothed (4-mm FWHM Gaussian kernel) by using the *Analysis of Functional NeuroImages* (AFNI) software [40].

### Within-Condition Functional Connectivity Patterns

Linear correlation analysis was implemented to compute the functional connectivity in each of the four resting-state conditions (the first eyes-closed session, and the EC, EO, and EO-F sessions). This process was done using the REST package. Briefly, two spheres (radius = 6 mm), one in the PCC (−5, −49, 40) and another in the MPFC (−1, 47, −4) were first defined for each participant in line with a previous study [14]. The averaged time course was then obtained from each sphere and the correlation analysis was performed in a voxel-wise way to generate the functional connectivity of the PCC and MPFC, called the PCC-FC map and the MPFC-FC map, respectively. Prior to the correlation analysis, a linear regression was performed to remove the effects of nine nuisance covariates: the global mean signal; the white matter signal [picked from (−30, 16, 23) in the white matter]; the cerebrospinal fluid signal [picked from (−5, −14, 23) in the lateral ventricle]; and six head motion parameters. To obtain the global mean time course, a whole-brain mask was created by removing the non-brain tissue in the anatomical images using the MRIcro software (by Chris Rorden, http://www.mricro.com, see original Ref. [41]). By removing the global signal, variances contributed by physiological artifacts are minimized since the global signal has been found to be associated with respiration-induced fMRI signal [42]. Removal of signals correlating with that in the ventricles and the white matter further reduces nonneuronal contributions to blood oxygen level-dependent (BOLD) correlations [14], [43]. Finally, the correlation coefficient maps were converted into *z* maps by Fisher's r-to-*z* transform to improve the normality [44]. To determine the within-group functional connectivity patterns, one-sample *t*-tests were performed on the individual *z* maps of the PCC-FC and the MPFC-FC, respectively. The within-condition statistical threshold was set at |*t*|>4.8975 (*P*<0.0001) and cluster size >135 mm^3^, which corresponds to a corrected *P*<0.0001. This correction was confined within the whole-brain mask (size: 1448118 mm^3^) and was determined by Monte Carlo simulations [45] that were performed by the program AlphaSim in AFNI (http://afni.nih.gov/afni/docpdf/AlphaSim.pdf).

### Generation of Midline ROIs

Given that the midline areas, including the PCC, PCu, MPFC and vACC, are the main components of the DMN (for reviews, see [6], [46], [47]), we therefore confined the between-condition comparisons to these midline ROIs to investigate the differences in a voxel-wise way. The functional connectivity results of the first eyes-closed resting-state session were used to generate the midline ROIs. The within-condition *t* maps (*t*>4.8975) of the PCC-FC and the MPFC-FC were summed into a single map in which the values of non-zero voxels were further set to one. This process yielded a binary map where we observed two biggest clusters covering the PCC/PCu and MPFC/vACC, respectively, which were further chosen as the midline ROIs of the DMN.

### Between-Condition Differences of Functional Connectivity

Paired *t*-tests were performed between any pairs of the three conditions on the individual *z* maps of the PCC-FC and the MPFC-FC (See *Within-Condition Functional Connectivity Patterns*), respectively. The comparisons were confined within the midline ROIs of the DMN in a voxel-wise way. The between-condition statistical threshold was set at |*t*|>2.093 (*P*<0.05) and cluster size >486 mm^3^, which corresponds to a corrected *P*<0.05. This correction was confined within the midline ROIs (size: 89208 mm^3^) and was determined by the Monte Carlo simulations [45] that were performed by the program AlphaSim in AFNI (http://afni.nih.gov/afni/docpdf/AlphaSim.pdf).

### Between-Condition Differences of the ALFF

The following procedure for calculating the ALFF was the same as that used in our previous studies [26], [27], and was implemented here using the REST package. Briefly, the time courses were first converted to the frequency domain using a Fast Fourier Transform (FFT). The square root of the power spectrum was calculated and then averaged across 0.01–0.08 Hz at each voxel. This averaged square root was taken as the ALFF. In order to reduce the global effects of variability across participants, as done in PET studies [4], the ALFF of each voxel was divided by the global mean ALFF value within the whole-brain mask obtained previously (see *Within-Condition Functional Connectivity Patterns* section). To reveal the between-condition differences of regional activities, paired *t*-tests were performed on the individual ALFF maps within the midline ROIs of the DMN in a voxel-wise way. The between-condition statistical threshold was set at |*t*|>2.093 (*P*<0.05) and cluster size >486 mm^3^, which corresponds to a corrected *P*<0.05. This correction was confined within the midline ROIs (size: 89208 mm^3^) and was determined by the Monte Carlo simulations [45] that were performed by the program AlphaSim in AFNI (http://afni.nih.gov/afni/docpdf/AlphaSim.pdf).

## Results

### Within-condition functional connectivity patterns

**Figure 1:**
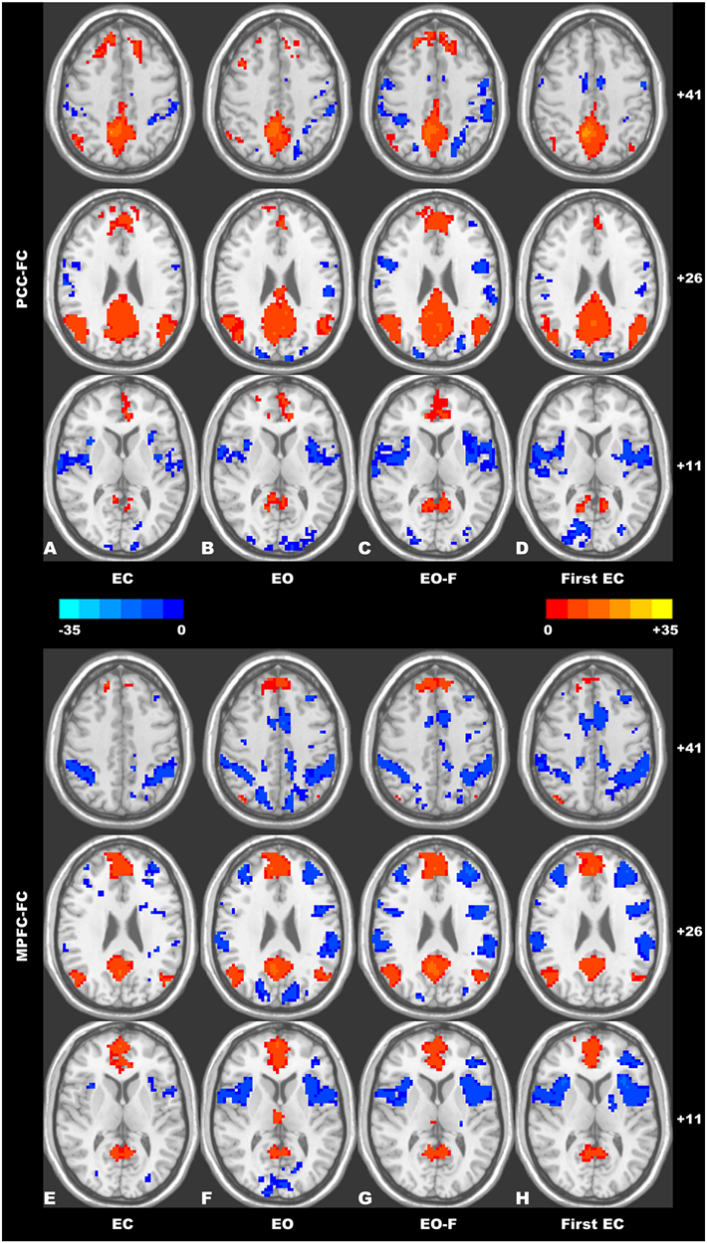
Within-condition functional connectivity patterns of the resting-state conditions: (A–D) the PCC-FC patterns for the EC, EO, EO-F, and the first EC conditions, respectively; (E–G) the MPFC-FC patterns for the EC, EO, EO-F, and the first EC conditions, respectively. The patterns of the DMN as well as its anti-correlated network were visually similar across the resting-state conditions. The numbers at the right side of the images refer to the z coordinates in the Talairach and Tournoux atlas. The statistical threshold was set at |*t*|>4.897 (*P*<0.0001) and cluster size >135 mm^3^, which corresponds to a corrected *P*<0.0001.

The one-sample *t*-tests revealed that the patterns of the DMN as well as its anti-correlated network were similar (spatial correlation coefficient between any two pairs of the *t* maps ranged from 0.86 to 0.92) across the resting-state conditions (i.e., the EC, EO, EO-F and the first EC condition) (Figure 1).

### Between-condition differences of functional connectivity

**Figure 2:**
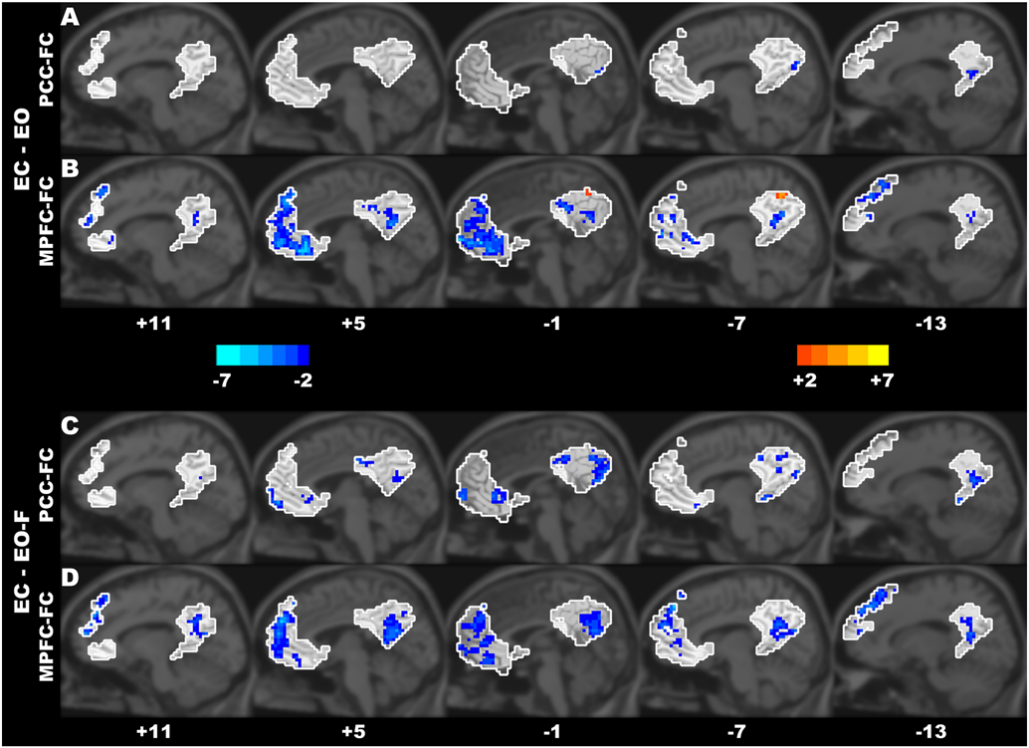
The between-condition differences of the functional connectivity within the DMN. Significant differences were found in the PCC-FC and the MPFC-FC maps between the EC and EO (A, B) and between the EC and EO-F (C, D) resting-state conditions. The areas in the white contours denote the ROIs within the DMN. The numbers below the images refer to the x coordinates in the Talairach and Tournoux space. The statistical threshold was set at |*t*|>2.093 (*P*<0.05) and cluster size >486 mm^3^, which corresponds to a corrected *P*<0.05.

Despite the highly similar patterns across the different resting-state conditions, the paired *t*-tests revealed significant between-condition differences for both the PCC-FC and the MPFC-FC maps within the midline ROIs of the DMN (*P*<0.05, corrected) (Figure 2, Figure S1, Table 1 and Table 2). In both the PCC-FC and the MPFC-FC maps, the PCC (Brodmann's area (BA) 23/31) showed a significantly decreased connectivity in the eyes-closed (i.e., EC) condition compared with the eyes-open (with and without fixation, i.e., EO-F and EO) conditions (Figure 2). In the MPFC-FC maps, the MPFC (BA 9/10) and the vACC (BA 32/24/25) showed a decreased connectivity in the eyes-closed (i.e., EC) condition compared with the eyes-open conditions (Figure 2B and 2D). In the PCC-FC maps, the MPFC (BA 9/10) and vACC (BA 32/24/25) showed a decreased connectivity in the EC compared with the EO-F conditions (Figure 2C). As mentioned above, the EC condition exhibited a decreased connectivity compared with the EO and EO-F conditions in most circumstances; however, in the MPFC-FC maps, a small part of the PCu (BA 7) showed an increased connectivity in the EC compared with the EO conditions (Figure 2B).

**Table 1:** Detailed information of the between-condition differences in the PCC-FC maps within the DMN.

Conditions	Connected regions	BA	Cluster volume (mm^3^)	*t*-score of peak voxel	Coordinates of peak voxel
EC - EO	L Cuneus/L PCu	18/31	486	−3.3504	−8,−76,26
	L PCC	30	486	−2.7665	−16,−56,12
EC - EO-F	Cuneus/PCu	18/31	3402	−4.6844	−4,−76,24
	vACC	25/24	1485	−3.4341	−2,34,2
	L PCC/L PCu	31	1377	−3.5702	−10,−56,30
	MPFC	10	1188	−5.5691	8,56,−4
	Cingulate Gyrus	31	1080	−3.6331	−4,−38,42
	L PHG/L Lingual Gyrus	30/19	486	−4.1661	−16,−44,-4
EO - EO-F	PCu	7	1674	−4.2299	−8,−62,42
	vACC	32	1566	−3.7358	−2,44,6
	MPFC/SFG	9	1188	−3.6885	−8,46,44

The statistical threshold was set at |*t*|>2.093 (*P*<0.05) and cluster size >486 mm^3^, which corresponds to a corrected *P*<0.05. PCC, posterior cingulate cortex; PCu, precuneus; MPFC, medial prefrontal cortex; vACC, ventral anterior cingulate cortex; SFG, superior frontal gyrus; PHG, parahippocampal gyrus; BA, Brodmann's area.

**Table 2:** Detailed information of the between-condition differences in the MPFC-FC maps within the DMN.

Conditions	Connected regions	BA	Cluster volume (mm^3^)	*t*-score of peak voxel	Coordinates of peak voxel
EC - EO	MPFC/vACC/SFG	11/10/32/24/9/8	16875	−6.8574	2,34,−10
	PCC/PCu	23/30/31	3483	−5.7841	−4,−62,26
	L SFG	8	1296	−3.7202	−16,28,56
	Cingulate Gyrus	31	1134	−3.7802	−2,−40,32
	L MPFC	9	999	−3.9344	−14,44,48
	L SFG	8	729	−4.3273	−28,20,60
	L PCu	7	486	4.0726	−8,−58,48
EC - EO-F	MPFC	9/10	13662	−5.3445	8,52,30
	PCC/PCu	23/31	9045	−4.1918	4,−62,20
	R SFG	8/9	1701	−5.284	16,32,48
	MPFC/vACC	11/10/32	1404	−3.5459	2,44,−10
EO - EO-F	vACC	25/24	1080	4.0116	−4,22,2
	L SFG	9/8	621	−3.5586	−10,46,44
	L Cuneus/L PCu	18/31	594	−4.1784	−8,−70,18
	R PCC	31	567	−3.7381	14,−64,18
	R MPFC/R SFG	9	486	−4.8718	8,50,30

The statistical threshold was set at |*t*|>2.093 (*P*<0.05) and cluster size >486 mm^3^, which corresponds to a corrected *P*<0.05. PCC, posterior cingulate cortex; PCu, precuneus; MPFC, medial prefrontal cortex; vACC, ventral anterior cingulate cortex; SFG, superior frontal gyrus; BA, Brodmann's area.

There were also significant differences between the eyes-open with and without fixation conditions (i.e., EO-F vs. EO). In the PCC-FC map, the MPFC (BA 9), vACC (BA 32/24/25), and PCu (BA 7) showed a decreased connectivity in the EO compared with the EO-F conditions (Figure S1A). In the MPFC-FC map, the PCC (BA 31) and MPFC (BA 9) showed a decreased connectivity in the EO compared with the EO-F conditions (Figure S1B). However, in the MPFC-FC map, the vACC (BA 25/24) showed an increased connectivity in the EO compared with the EO-F condition (Figure S1B).

### Between-condition differences of the ALFF

**Figure 3:**
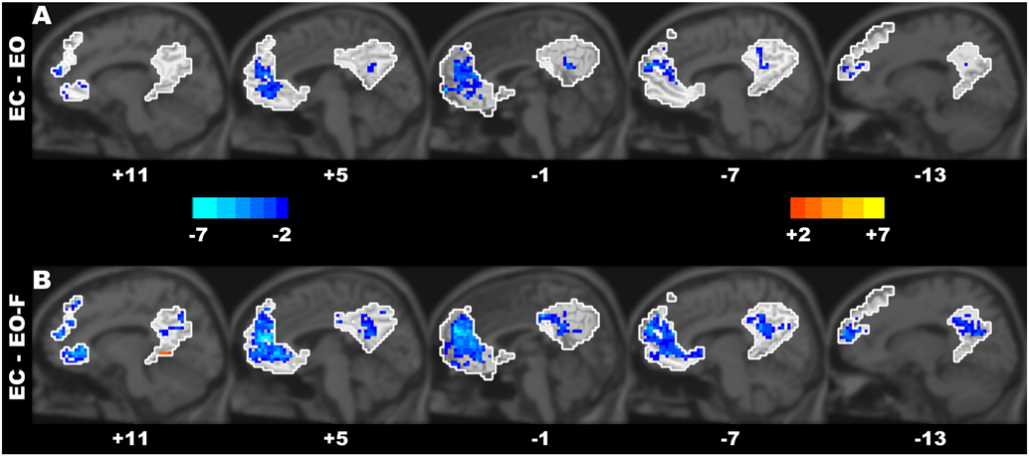
The between-condition differences of the ALFF within the DMN. The ALFF differences were found between the EC and EO conditions (A), and between the EC and EO-F conditions (B). The areas in the white contours denote the ROIs within the DMN. The numbers below the images refer to the x coordinates in the Talairach and Tournoux space. The statistical threshold was set at |*t*|>2.093 (*P*<0.05) and cluster size >486 mm^3^, which corresponds to a corrected *P*<0.05.

The paired *t*-tests revealed significant between-condition differences in the ALFF within the midline ROIs of the DMN (*P*<0.05, corrected) (Figure 3, Figure S1, and Table 3). The PCC (BA 23/31) and the MPFC (BA 9/10) showed significantly decreased ALFF in the eyes-closed (i.e., EC) condition compared with the eyes-open conditions (Figure 3). However, a small part of the parahippocampal gyrus (BA 30/19) showed increased ALFF in the EC compared with EO-F conditions (Figure 3B). In addition, we also noted that the MPFC (BA 10/9), vACC (BA 32), and PCu (BA 7) showed decreased ALFF in the EO compared with the EO-F conditions (Figure S1C).

**Table 3:** Detailed information of the between-condition differences of the ALFF within the DMN.

Conditions	Connected regions	BA	Cluster volume (mm^3^)	*t*-score of peak voxel	Coordinates of peak voxel
EC - EO	MPFC/vACC	10/9/24/32	10773	−5.1453	−2,64,2
	PCC	23/31	1431	−3.5878	−4,−52,24
EC - EO-F	vACC/MPFC	32/24/10/9	22707	−7.2085	2,50,14
	PCC/PCu	31/7	9234	−4.9118	−2,−28,38
	R SFG	8	648	−3.0617	16,26,54
	R PHG/R Lingual Gyrus	30/19	486	4.1819	16,−46,2
	R SFG	8	486	−4.1525	10,38,50
EO - EO-F	PCu/PCC	7/23/31	2565	−3.4712	−4,−64,32
	MPFC	10/9	1755	−5.0419	2,56,32
	MPFC/vACC	11/10/24/32	1728	−4.2298	8,32,0
	MPFC	32/9	756	−4.4373	10,40,30

The statistical threshold was set at |*t*|>2.093 (*P*<0.05) and cluster size >486 mm^3^, which corresponds to a corrected *P*<0.05. PCC, posterior cingulate cortex; PCu, precuneus; MPFC, medial prefrontal cortex; vACC, ventral anterior cingulate cortex; SFG, superior frontal gyrus; PHG, parahippocampal gyrus; BA, Brodmann's area.

## Discussion

Using resting-state fMRI, the current study compared spontaneous brain activity within the DMN among different resting-state conditions (i.e., EC, EO, and EO-F) from both the integrative (FC) and the regional (ALFF) perspectives. Although the connectivity maps were visually similar across the three resting-state conditions, the strength of functional connectivity and the ALFF showed significant differences between the conditions, thus providing quantitative evidence to support the idea that the spontaneous brain activity in the DMN is associated with different resting conditions with limited cognitive load.

### Consistency of the DMN patterns among various resting conditions

In this study, we observed that the DMN patterns, as well as its anti-correlated network patterns, detected by the functional connectivity analysis were visually similar across the resting-state conditions (EC, EO, EO-F and the first EC condition, see Figure 1), which is consistent with previous resting-state fMRI studies [5], [14], [15]. In general, these studies found that the spontaneous fluctuations among the regions within the DMN were temporally coherent during various awake resting-states [5], [14], [15], during task performance [25], and during light sleep [21]. These consistent results indicate that, under different resting-state conditions, the brain areas in the DMN might be commonly involved in unconstrained mental activities like self-reflective thought, monitoring of the environment, and daydreaming [6], [11], [25].

### Differences within the DMN among various resting conditions (EC, EO and EO-F)

Although previous studies have suggested that the DMN patterns are similar across multiple resting-state conditions and are minimally disturbed across these conditions, no statistical comparisons have been performed to examine the condition-associated differences in the connectivity maps [5], [14], [15]. Therefore, it remains unclear whether the spontaneous brain activity could be changed by different resting states. It has been reported that, during a sustained working memory condition, the functional connectivity within the DMN was significantly lower than that during the resting visual fixation condition [25]. A statistical comparison would thus help us to find the subtle differences between different resting states. The current study found significant differences of functional connectivity among the three resting-state conditions (i.e., EC, EO, and EO-F). Most regions of the PCC and the MPFC within the DMN (Figure 2) in both the EO and the EO-F exhibited higher functional connectivity than that in the EC condition. These results suggest that the DMN is more highly synchronized during the eyes-open (both with and without fixation) states than during the eyes-closed conditions. It has been suggested that the PCC is associated with the general monitoring of sensory information and that the MPFC is associated with an evaluation of the salience of this information [4], [6]. When the eyes are kept open, especially in a new environment, more non-specific or non-goal-directed information may be automatically and continuously gathered and evaluated. This process is possibly associated with more coherent activities within the DMN. Recent studies have also suggested that the DMN, including the PCC and the MPFC, is related to mind wandering and daydreaming [11]. We tentatively suggest that, during the eyes-open conditions, the increased information gathering and evaluation may also be associated with increased mind wandering or daydreaming, leading to more tightly coherent activities among the regions in the DMN.

The functional connectivity analysis can reveal the difference in synchronization among brain areas between different resting-state conditions; however, it does not reflect the differences of regional brain activities. The regional spontaneous activities can be examined by the ALFF. Of note, the mechanism of the LFF BOLD signal was currently unclear. Biswal et al. [18] found that the root mean square of the LFF in the white matter was reduced by about 60% relative to the gray matter. The power spectrum of the LFF (equivalent to the square of the amplitude of the LFF) has been used to indicate the magnitude of neural activity [24], [25]. Logothetis et al. [19] found that the local field potential yields a good estimate of the BOLD responses, suggesting that the regional spontaneous BOLD signal may reflect spontaneous neural activities. Thus, it has been speculated that ALFF could be an index for measuring the amplitude of regional spontaneous neuronal activity [26], [27]. Using the ALFF, the current study found that there were significantly higher ALFF in the PCC and the MPFC in both of the EO and EO-F conditions, as compared to the EC condition (Figure 3). This result is compatible with our results from the functional connectivity analysis. As mentioned above, the brain areas within the DMN have been suggested to be involved in monitoring the environment and gathering information around us [4], [6], as well as mind wandering and daydreaming [11]. Our recent studies have suggested that the ALFF might be a measure of the spontaneous neuronal activity in brain regions [26], [27] associated with the low-frequency BOLD fluctuations obtained by resting fMRI [19], [48]. Thus, our findings of higher ALFF within the DMN might reflect increased spontaneous neuronal activities in the related regions due to a broad information gathering and evaluation, and mind wandering and daydreaming when the eyes keep open (i.e., EO and EO-F conditions).

In addition to the well-accepted midline areas within the DMN, the inferior parietal lobule (IPL, i.e., BA 39 and 40) is also an important area in the DMN [6], [46], [47]. We further compared the functional connectivity (i.e., the Z map) and ALFF of the IPL among different conditions (See Text S1 and Figure S2 for details). Compared with the EC, both the EO and the EO-F conditions exhibited higher functional connectivity and ALFF in the IPL (Figure S3), which was consistent with our findings in the MPFC and the PCC (Figure 2 and Figure 3). A previous study [6] suggests that the IPL is obligatorily or unintentionally engaged in the recall of episodic memory information. Thus, we speculate that there might be more episodic memory information recalled when the eyes keep open than closed, which needs to be clarified in the future. Together, our results provide empirical evidence that the spontaneous activity in the DMN is sensitive to the changes of conditions, even with a low cognitive load.

Our findings of spontaneous brain activity in the DMN associated with different resting conditions raised the issue of what kind of condition is more appropriate for clinical studies. To date, several different resting-state conditions have been used in the patient's studies [30]–[37]. For example, in the research area of attention-deficit/hyperactivity disorder, some studies used the eyes-open condition [35], [36], while others used the eyes-closed condition [37]. Considering that we showed significant differences of the spontaneous activity in the DMN between the eyes-open and the eyes-closed conditions, we therefore suggest it should be cautious when comparing the results across different studies in which different resting-state conditions are used. In future studies, it will be very important to determine a standard resting-state condition for clinical fMRI studies.

### The order effect of scanning sessions: the first EC and the second EC

**Figure 4:**
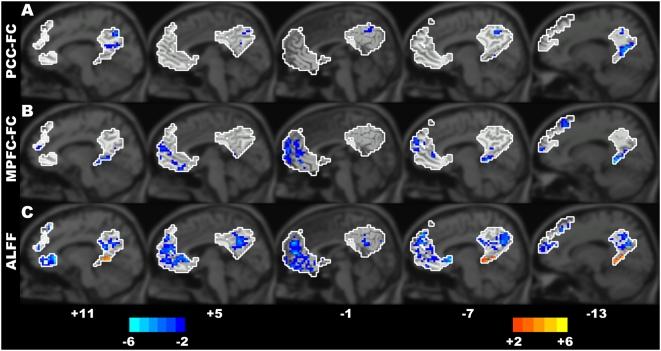
The differences in the PCC-FC maps (A), the MPFC-FC maps (B), and the ALFF maps (C) between the first EC condition and the second EC condition (First EC - Second EC) within the DMN. The areas in the white contours denote the ROIs within the DMN. The numbers below the images refer to the x coordinates in the Talairach and Tournoux atlas. The statistical threshold was set at |*t*|>2.093 (*P*<0.05) and cluster size >486 mm^3^, which corresponds to a corrected *P*<0.05.

The aforementioned differences were found between different resting conditions. Furthermore, a visual inspection of Figure 1 suggests apparent differences between the first and second EC conditions. To assess whether there is an order effect on the DMN, we compared the differences in the DMN between the first and second EC conditions in the same way used in the assessment of the between-condition differences. We found that several key regions of the DMN (including the MPFC, PCC and IPL) had higher ALFF and functional connectivity in the second EC than in the first one (Figure 4 and Figure S4). Previous studies have suggested that the DMN is involved in episodic memory consolidation and retrieval [6], [49], [50]. In the current study, there were several events (e.g., the EO, EO-F and an event-related visual task condition) between the first EC and the second EC conditions for most participants since the four conditions (EO, EO-F, the event-related visual task and the second EC conditions) were counterbalanced across participants after the first EC condition (See Methods for details). Thus, the increased spontaneous activity of the DMN in the second EC might reflect an increased episodic memory consolidation or retrieval. However, this assumption needs to be tested in the future since very few studies reported the relationship between the spontaneous neural activity of the human brain and memory consolidation. The current findings might also be helpful for the experimental design of the resting-state fMRI studies. Although a few studies found that the connectivity patterns in resting brains were remarkably consistent across sessions acquired at different time (from minutes apart to months apart) [51], [52], our results by direct comparison indicate that the activity within the DMN was not only modulated by different resting conditions but also by the order of the same type of resting condition (i.e., EC). The results of the order effect also suggest that it should be cautious when designing which run the resting-state condition should be placed in multiple scanning sessions.

### Differences of other brain regions among various resting conditions

In addition to revealing the functional changes within the DMN across different resting-state conditions, investigating the functional change of other brain regions may also be helpful. From the whole brain comparisons (Figure S5, Figure S6, Figure S7 and Figure S8), we found significant differences in the ALFF across different conditions in the visual cortex. For example, there was significantly higher ALFF (P<0.05, corrected) in the visual cortex in the EO condition than in the EC condition (Figure S5, arrow 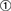), which was consistent with Ref. [27]. The higher activity of the visual cortex in the EO condition could be due to that this region was activated by visual input when the participants kept their eyes open.

We also found that some regions within the anti-correlation network showed significant differences across different conditions (Figure S5, Figure S6, Figure S7 and Figure S8). For example, there was significantly lower ALFF (P<0.05, corrected) in the left insula in the EO condition than the EC condition (Figure S5, arrow 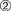). Previous studies have suggested a competitive relationship between the DMN and the anti-correlated network, and that these two networks may serve differentiating roles in segregating neuronal processes subserving opposite goals [14], [15]. The higher activity in the DMN and the lower activity in the anti-correlated network in the EO condition also indicated a dynamic balance between the two networks. The functional significance of such a change in this network needs to be further investigated in the future.

### Concerns

One concern is the functional complexity of the DMN. We found that the human spontaneous brain activity in the DMN could be easily modulated, but the psychophysiological mechanisms underlying the modulation need to be further investigated in the future.

### Conclusions

In summary, this study demonstrated that both the functional connectivity and the ALFF within the DMN were significantly different across various resting-state conditions, suggesting that the DMN is sensitive to perturbations even with limited cognitive demand. These condition-related differences might be due to that the resting state with the eyes open is associated with more non-specific or non-goal-directed visual information gathering and evaluating, as well as mind wandering and daydreaming compared with the eyes closed resting state. In addition, the spontaneous neuronal activity within the DMN may also be modulated by the order of scanning, even with the same type of resting condition. These results also suggest that it should be cautious when choosing the type of a resting condition and designating the order of the resting condition in multiple scanning sessions in experimental design. This study also has implications for our understanding of the functional complexity of the human DMN.

## Supporting Information

Figure S1 The differences in the PCC-FC maps (A), the MPFC-FC maps (B), and the ALFF maps (C) between the EO and the EO-F (EO - EO-F) conditions within the DMN. The areas in the white contours denote the ROIs within the DMN. The numbers below the images refer to the x coordinates in the Talairach and Tournoux atlas. The statistical threshold was set at |*t*|>2.093 (*P*<0.05) and cluster size >486 mm^3^, which corresponds to a corrected *P*<0.05.
(0.22 MB JPG)

Figure S2 The PCC functional connectivity (A) and MPFC functional connectivity (B) patterns within BA 39/40 of the first EC condition. The functional connectivity with the PCC or MPFC in the first EC condition showed that there were functional segregations in the IPL (BA 39/40). The numbers below the images refer to the z coordinates in the Talairach and Tournoux atlas. The statistical threshold was set at |*t*|>4.897 (*P*<0.0001, uncorrected).
(0.50 MB JPG)

Figure S3 The between-condition differences within the IPL [regions that showed significant positive functional connectivity (t>4.897, P<0.0001, uncorrected) with the PCC or the MPFC within BA 39/40)] of different resting-state conditions: (A–C) the PCC-FC, MPFC-FC, and ALFF differences between the EC and EO (EC - EO) conditions, respectively; (D–F) the PCC-FC, MPFC-FC, and ALFF differences between the EC and EO-F (EC - EO-F) conditions, respectively; (G–I) the PCC-FC, MPFC-FC, and ALFF differences between the EO and EO-F (EO - EO-F) conditions, respectively. The numbers below the images refer to the z coordinates in the Talairach and Tournoux atlas. The statistical threshold was set at |*t*|>2.093 (*P*<0.05, uncorrected).
(1.12 MB JPG)

Figure S4 The differences in the PCC-FC maps (A), the MPFC-FC maps (B), and the ALFF maps (C) between the first EC condition and the second EC condition (First EC - Second EC) within the IPL [regions that showed significant positive functional connectivity (t>4.897, P<0.0001, uncorrected) with the PCC or MPFC within BA 39/40)]. The numbers below the images refer to the z coordinates in the Talairach and Tournoux atlas. The statistical threshold was set at |*t*|>2.093 (*P*<0.05, uncorrected).
(0.41 MB JPG)

Figure S5The differences in the PCC-FC maps (A), the MPFC-FC maps (B), and the ALFF maps (C) between the EC and EO (EC - EO) conditions. There was a significantly higher ALFF in the visual cortex in the EO condition than in the EC condition (arrow 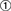), and a significantly lower ALFF in the left insula (within the anti-correlated network) in the EO condition than in the EC condition (arrow 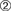). The numbers at the lower right corner of the images refer to the z coordinates in the Talairach and Tournoux atlas. The statistical threshold was set at |*t*|>2.093 (*P*<0.05) and cluster size >1431 mm^3^, which corresponds to a corrected *P*<0.05.(0.87 MB JPG)

Figure S6 The differences in the PCC-FC maps (A), the MPFC-FC maps (B), and the ALFF maps (C) between the EC and the EO-F (EC - EO-F) conditions. The numbers at the lower right corner of the images refer to the z coordinates in the Talairach and Tournoux atlas. The statistical threshold was set at |*t*|>2.093 (*P*<0.05) and cluster size >1431 mm^3^, which corresponds to a corrected *P*<0.05.
(0.93 MB JPG)

Figure S7 The differences in the PCC-FC maps (A), the MPFC-FC maps (B), and the ALFF maps (C) between the EO and the EO-F (EO - EO-F) conditions. The numbers at the lower right corner of the images refer to the z coordinates in the Talairach and Tournoux atlas. The statistical threshold was set at |*t*|>2.093 (*P*<0.05) and cluster size >1431 mm^3^, which corresponds to a corrected *P*<0.05.
(0.78 MB JPG)

Figure S8 The differences in the PCC-FC maps (A), the MPFC-FC maps (B), and the ALFF maps (C) between the first EC condition and the second EC condition (First EC - Second EC). The numbers at the lower right corner of the images refer to the z coordinates in the Talairach and Tournoux atlas. The statistical threshold was set at |*t*|>2.093 (*P*<0.05) and cluster size >1431 mm^3^, which corresponds to a corrected *P*<0.05.
(0.93 MB JPG)

Text S1 The differences within the IPL across different resting conditions.
(0.03 MB DOC)
