# Supplementary figures and images for: Spontaneous Brain Activity in the Default Mode Network Is Sensitive to Different Resting-State Conditions with Limited Cognitive Load
Source: PLoS One. 2009 May 29;4(5):e5743. doi: 10.1371/journal.pone.0005743 (PMC2683943; doi:10.1371/journal.pone.0005743)

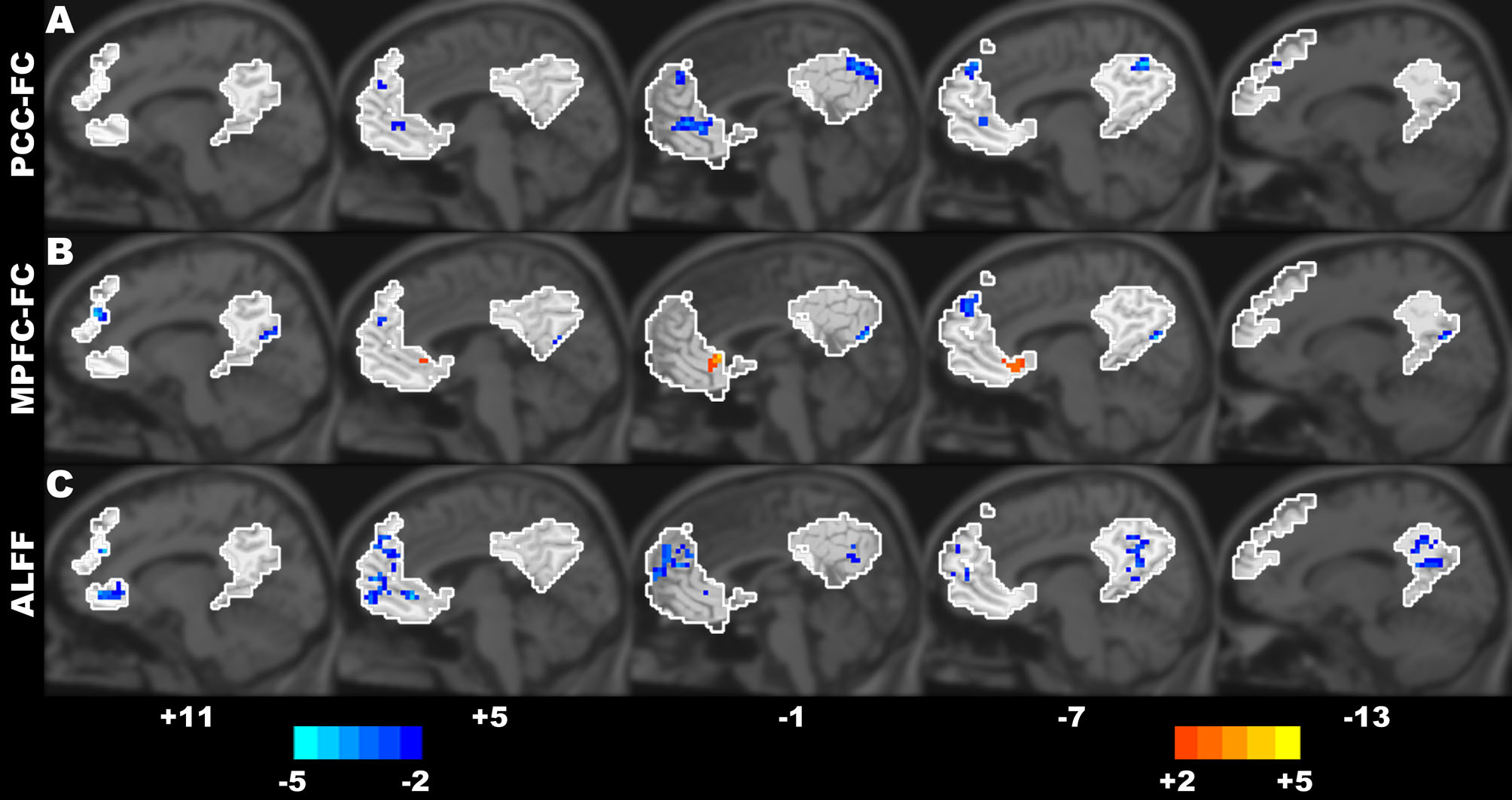

Supplement: Figure S1 — The differences in the PCC-FC maps (A), the MPFC-FC maps (B), and the ALFF maps (C) between the EO and the EO-F (EO - EO-F) conditions within the DMN. The areas in the white contours denote the ROIs within the DMN. The numbers below the images refer to the x coordinates in the Talairach and Tournoux atlas. The statistical threshold was set at |t|>2.093 (P<0.05) and cluster size >486 mm3, which corresponds to a corrected P<0.05. (0.22 MB JPG) [file pone.0005743.s001.jpg]

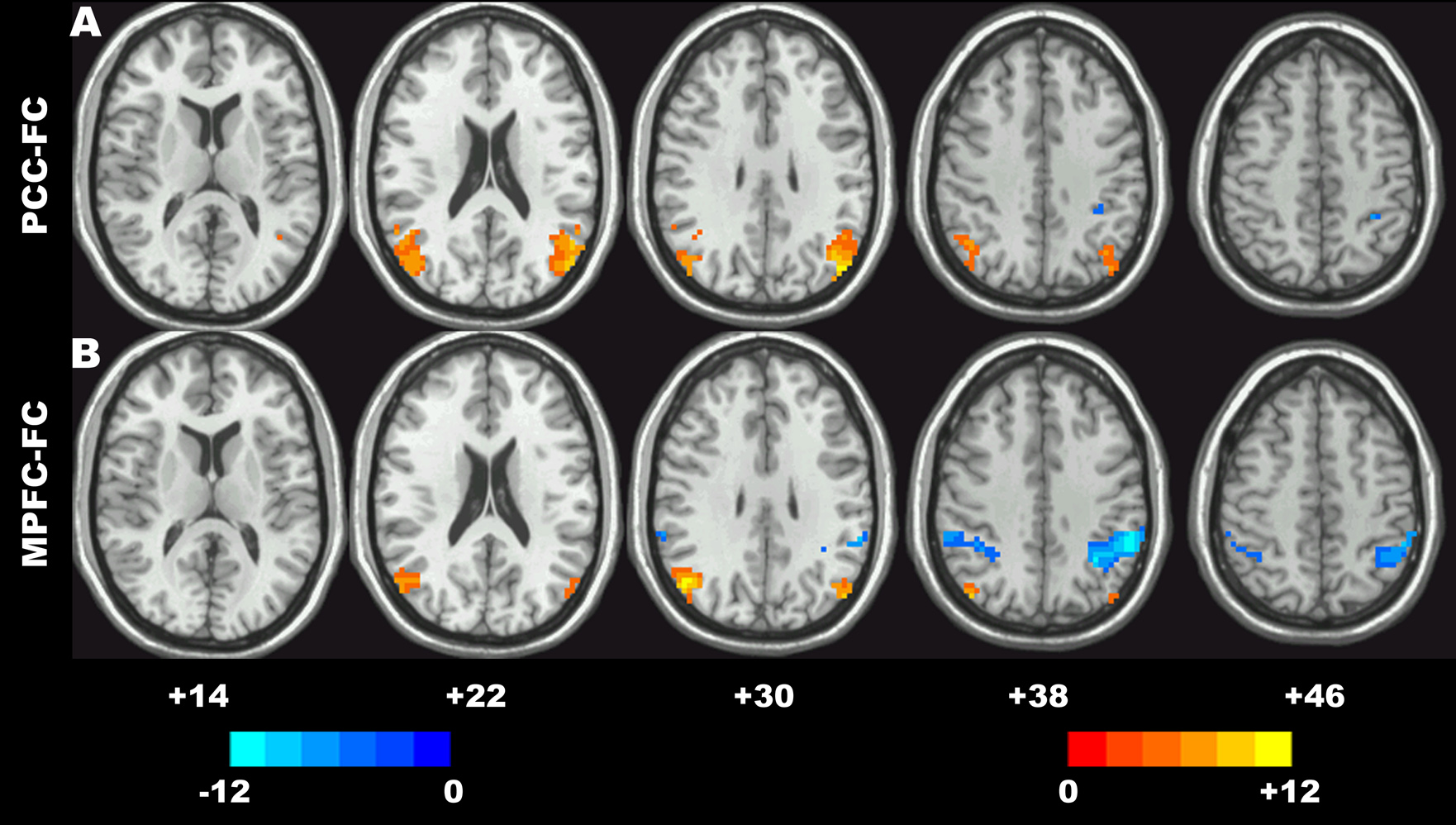

Supplement: Figure S2 — The PCC functional connectivity (A) and MPFC functional connectivity (B) patterns within BA 39/40 of the first EC condition. The functional connectivity with the PCC or MPFC in the first EC condition showed that there were functional segregations in the IPL (BA 39/40). The numbers below the images refer to the z coordinates in the Talairach and Tournoux atlas. The statistical threshold was set at |t|>4.897 (P<0.0001, uncorrected). (0.50 MB JPG) [file pone.0005743.s002.jpg]

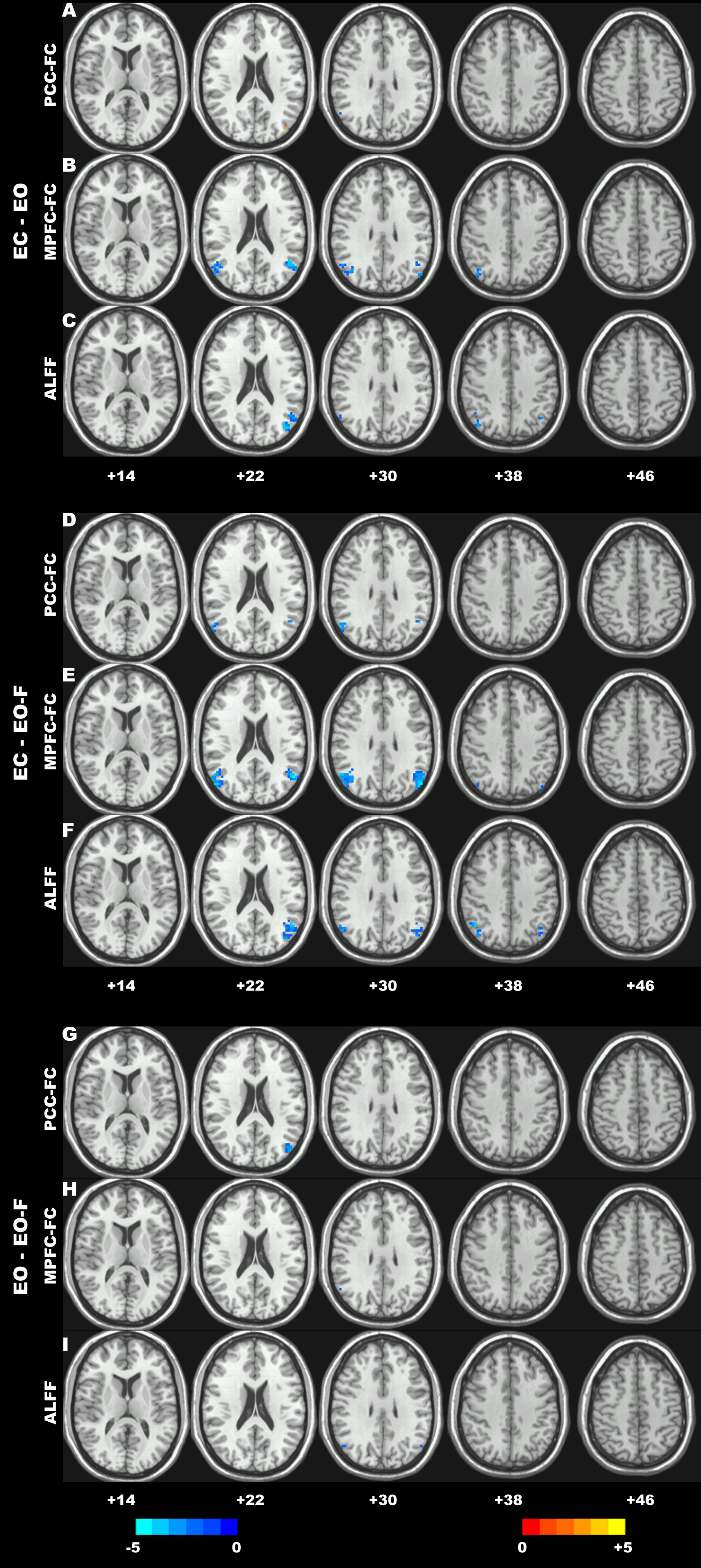

Supplement: Figure S3 — The between-condition differences within the IPL [regions that showed significant positive functional connectivity (t>4.897, P<0.0001, uncorrected) with the PCC or the MPFC within BA 39/40)] of different resting-state conditions: (A–C) the PCC-FC, MPFC-FC, and ALFF differences between the EC and EO (EC - EO) conditions, respectively; (D–F) the PCC-FC, MPFC-FC, and ALFF differences between the EC and EO-F (EC - EO-F) conditions, respectively; (G–I) the PCC-FC, MPFC-FC, and ALFF differences between the EO and EO-F (EO - EO-F) conditions, respectively. The numbers below the images refer to the z coordinates in the Talairach and Tournoux atlas. The statistical threshold was set at |t|>2.093 (P<0.05, uncorrected). (1.12 MB JPG) [file pone.0005743.s003.jpg]

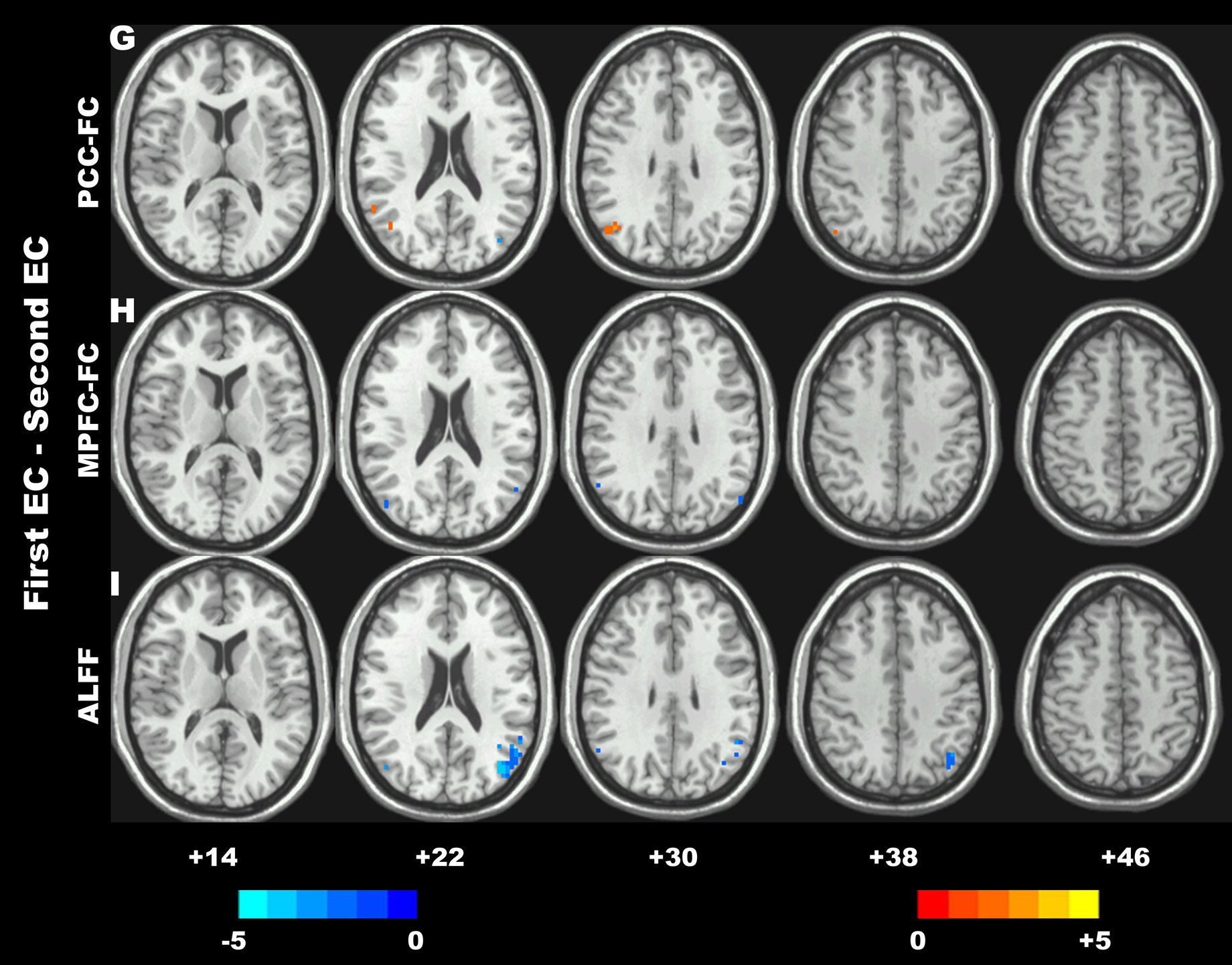

Supplement: Figure S4 — The differences in the PCC-FC maps (A), the MPFC-FC maps (B), and the ALFF maps (C) between the first EC condition and the second EC condition (First EC - Second EC) within the IPL [regions that showed significant positive functional connectivity (t>4.897, P<0.0001, uncorrected) with the PCC or MPFC within BA 39/40)]. The numbers below the images refer to the z coordinates in the Talairach and Tournoux atlas. The statistical threshold was set at |t|>2.093 (P<0.05, uncorrected). (0.41 MB JPG) [file pone.0005743.s004.jpg]

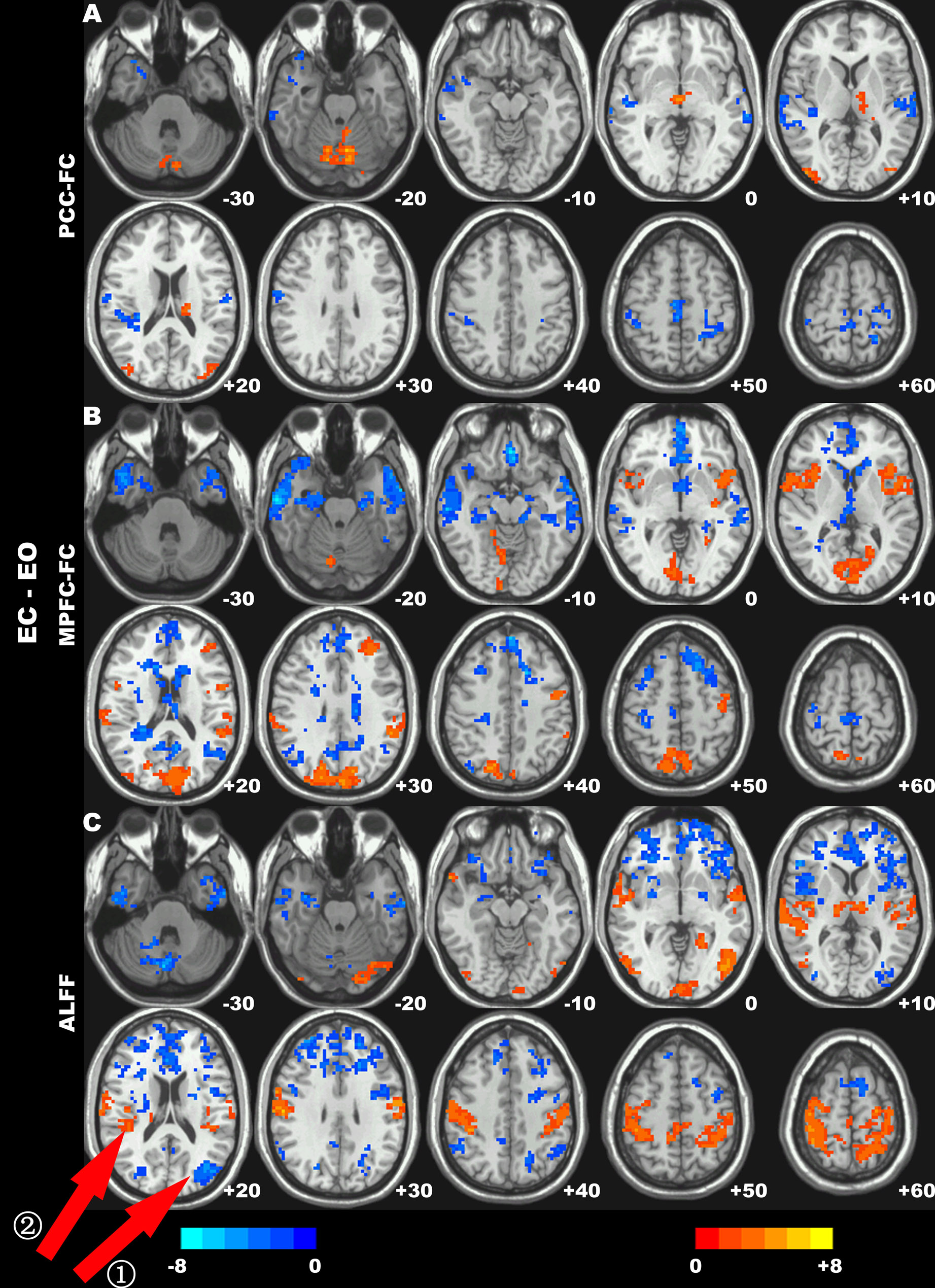

Supplement: Figure S5 — The differences in the PCC-FC maps (A), the MPFC-FC maps (B), and the ALFF maps (C) between the EC and EO (EC - EO) conditions. There was a significantly higher ALFF in the visual cortex in the EO condition than in the EC condition (arrow ), and a significantly lower ALFF in the left insula (within the anti-correlated network) in the EO condition than in the EC condition (arrow ). The numbers at the lower right corner of the images refer to the z coordinates in the Talairach and Tournoux atlas. The statistical threshold was set at |t|>2.093 (P<0.05) and cluster size >1431 mm3, which corresponds to a corrected P<0.05. (0.87 MB JPG) [file pone.0005743.s005.jpg]

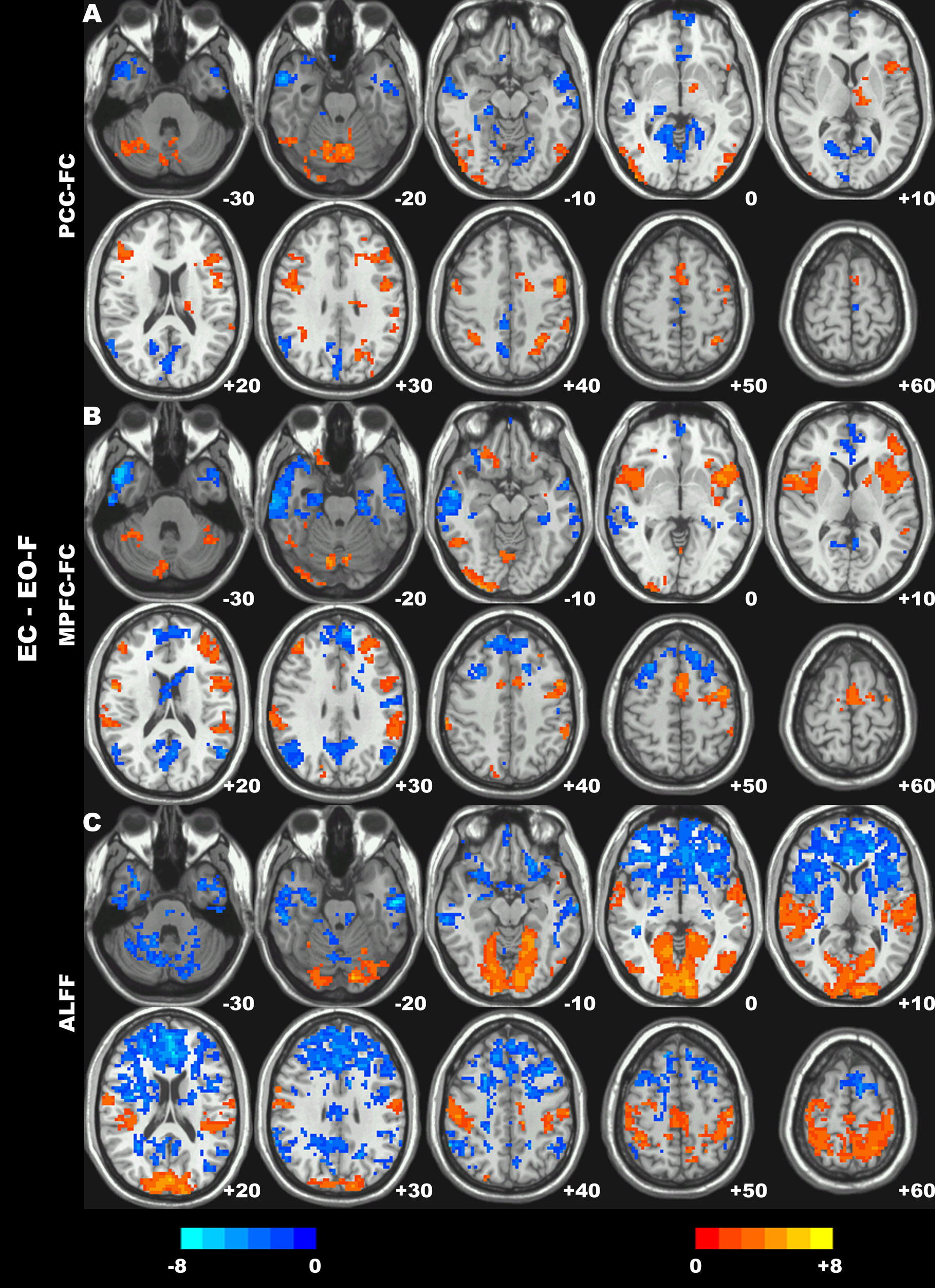

Supplement: Figure S6 — The differences in the PCC-FC maps (A), the MPFC-FC maps (B), and the ALFF maps (C) between the EC and the EO-F (EC - EO-F) conditions. The numbers at the lower right corner of the images refer to the z coordinates in the Talairach and Tournoux atlas. The statistical threshold was set at |t|>2.093 (P<0.05) and cluster size >1431 mm3, which corresponds to a corrected P<0.05. (0.93 MB JPG) [file pone.0005743.s006.jpg]

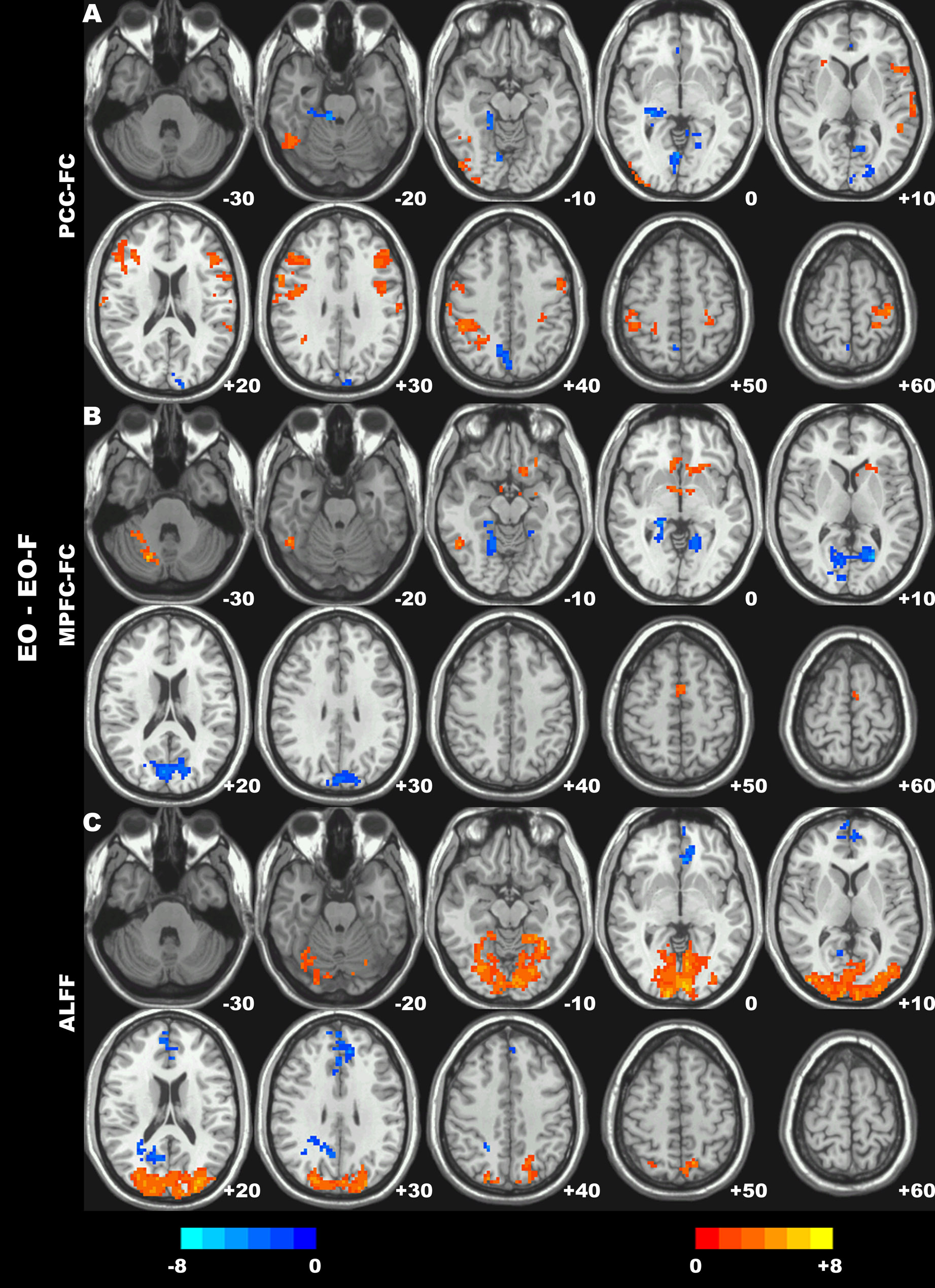

Supplement: Figure S7 — The differences in the PCC-FC maps (A), the MPFC-FC maps (B), and the ALFF maps (C) between the EO and the EO-F (EO - EO-F) conditions. The numbers at the lower right corner of the images refer to the z coordinates in the Talairach and Tournoux atlas. The statistical threshold was set at |t|>2.093 (P<0.05) and cluster size >1431 mm3, which corresponds to a corrected P<0.05. (0.78 MB JPG) [file pone.0005743.s007.jpg]

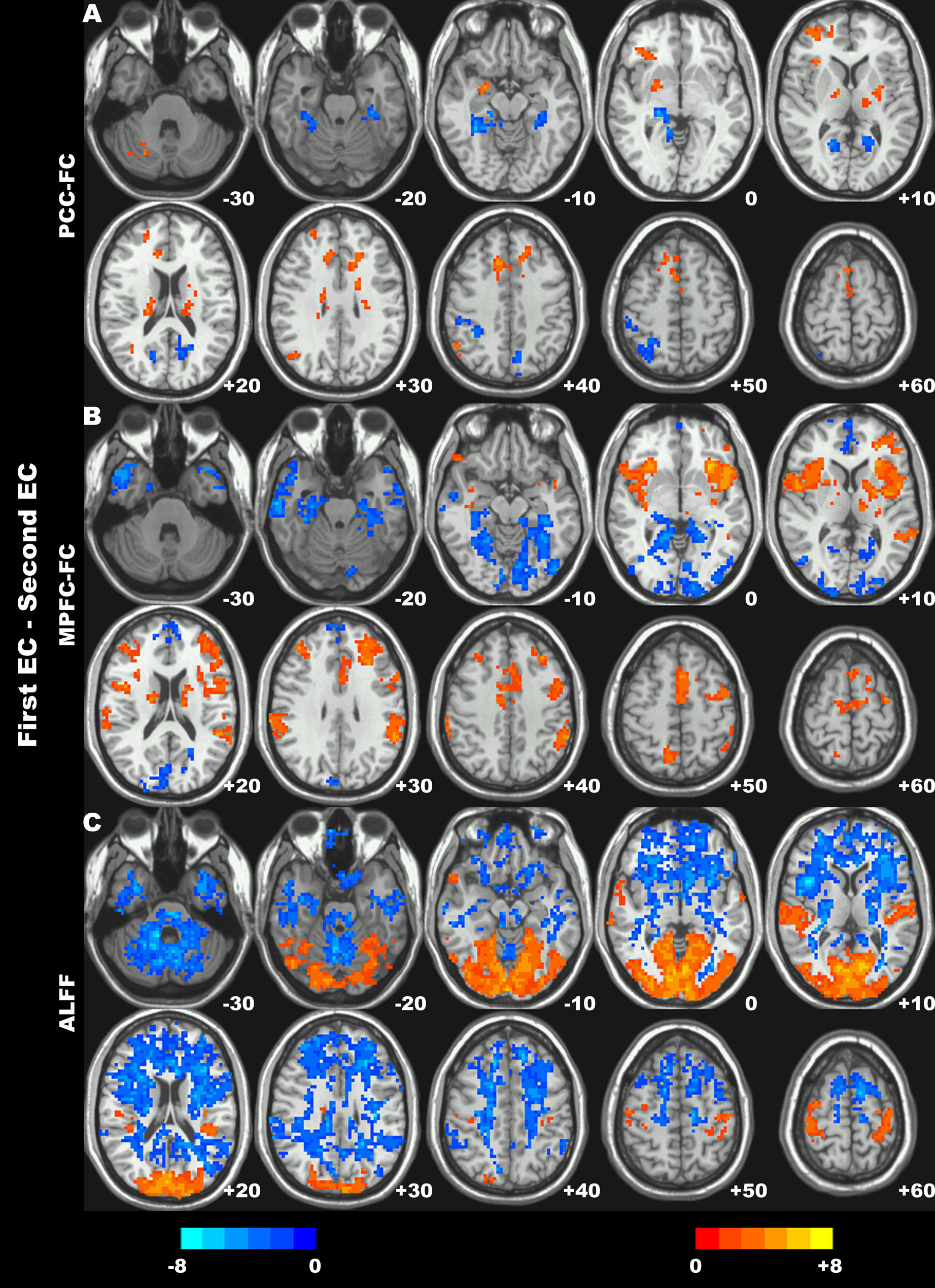

Supplement: Figure S8 — The differences in the PCC-FC maps (A), the MPFC-FC maps (B), and the ALFF maps (C) between the first EC condition and the second EC condition (First EC - Second EC). The numbers at the lower right corner of the images refer to the z coordinates in the Talairach and Tournoux atlas. The statistical threshold was set at |t|>2.093 (P<0.05) and cluster size >1431 mm3, which corresponds to a corrected P<0.05. (0.93 MB JPG) [file pone.0005743.s008.jpg]
