# Supplementary material for: Spontaneous Brain Activity in the Default Mode Network Is Sensitive to Different Resting-State Conditions with Limited Cognitive Load
Source: PLoS One. 2009 May 29;4(5):e5743. doi: 10.1371/journal.pone.0005743 (PMC2683943; doi:10.1371/journal.pone.0005743)
Supplement: Text S1 — The differences within the IPL across different resting conditions. (0.03 MB DOC) [file pone.0005743.s009.doc]

By investigating the functional connectivity patterns of the IPL (BA 39 and 40. From Brodmann template in MRIcro software by Chris Rorden; http://www.mricro.com) in the first EC condition, we found that the IPL showed both positive and negative functional connectivity with the MPFC or the PCC (Figure S2), suggesting that the IPL is functionally heterogeneous. We therefore focused only on the regions showing positive functional connectivity with the MPFC or the PCC in the BA 39 and 40 to investigate the differences across different conditions. Paired *t*-tests were performed between any pairs of the EC, EO and EO-F conditions on the PCC-FC, MPFC-FC and the ALFF map (obtained previously, see Methods for more details), respectively, within the IPL regions of interest in a voxel-wise way. The between-condition statistical threshold was set at |*t*| > 2.093 (*P* < 0.05, uncorrected).
